# Temperature Optimization by Using Response Surface Methodology and Desirability Analysis of Aluminium 6061

**DOI:** 10.3390/ma15175892

**Published:** 2022-08-26

**Authors:** Endalkachew Mosisa Gutema, Mahesh Gopal, Hirpa Gelgele Lemu

**Affiliations:** 1 Department of Mechanical Engineering, College of Engineering and Technology, Wollega University, Nekemte P.O. Box 395, Ethiopia; 2 Department of Mechanical and Structural Engineering and Materials Science, Faculty of Science and Technology, University of Stavanger, N-4036 Stavanger, Norway

**Keywords:** aluminium, cutting speed, response surface methodology, rate of feed, ANOVA, desirability function, cutting depth, tool nose radius

## Abstract

Because aluminium is a lightweight and low-density material, its alloys, such as Al 6061 alloy, are extensively used in numerous automobile, defense, and aviation components. This study aims to develop a predictive model to investigate the impact of tool nose radius on the CNC turning process of Al 6061 alloy and better recognize the implications of operating machining considering cutting speed, rate of feed, cutting depth, and tool nose radius. The trials were carried out by using the response surface methodology (RSM), with an Al_2_O_3_ coated carbide tool as the cutter and an Al 6061 workpiece as the material. A mathematical model of the second-order was created. The analysis of variance (ANOVA) approach was used to analyze the performance characteristics of the turning operation. Individual desirability values from the desirability function analysis for the multi-responses are used to construct a composite desirability value. The ideal parameter levels were determined by using the composite desirability value, and the significant impact of parameters was assessed by using the analysis of variance. The minimum temperature attained at the machining parameters are 98.0 m/min cutting speed, 0.26 mm/rev rate of feed, 0.893 mm cutting depth, and 0.84 mm tool nose radius. The best total desirability value is 23.615 °C, indicating that the experimental results are close to the predicted values.

## 1. Introduction

Aluminium is perhaps the most abundant element and the most common metal, contributing to 8% of the earth’s mantle. Due to their excellent mechanical qualities and low weight, aluminium alloys are increasingly being used as building elements in metal matrix composite (MMC) materials. The addition of metal reinforcements, such as aluminium alloys in MMCs makes machining of the composite harder and more difficult to predict the machining performance [1]. The tremendous degrees of temperature that are generated while machining increases tool wear, reduces tool life, and causes poor surface finish. As a result, previous studies have focused on improving the machining process of MMCs, particularly aluminium alloy-reinforced composites, using both experimental studies [2,3] and developing different prediction and optimization models [4] by considering factors such as machining process, type of equipment, the cutting tools, cutting speeds, feed rates, and lubricants.

Much material research has been directed toward developing innovative industrial materials of high strength-to-weight proportions, relatively high strengths, high thermal stiffness, and improved creep, endurance, and fatigue strength. In particular, improved material performance is required for sophisticated aerospace-sector technology [5]. The apparently abrasive nature of the particles, which function as a cutting edge during machining of hybrid MMC, is problematic and results in rapid wear of the tool and causes vibration. In the work reported in [6], experimental examination was done by using LM6 aluminium alloy material in the turning process to predict surface roughness, vibrations, and tool wear. The ANOVA and RSM methodology used to analyze the cutting characteristics showed that the resulting tool interface temperature was 51.75 °C. A drilling operation was performed by using aluminum silicon carbide [7] to measure MRR and surface roughness. The Taguchi technique is used to create the statistical equation. The ANOVA and MOGA optimization techniques were used to enhance the cutting performance. The author in [8] used the DFA analysis to determine the surface quality and cutting parameter force performance index. RSM and central composite design (CCD) methodology is used in investigations using the Al 356 alloy reinforced with SiC. The results show that a better surface finish was obtained at higher cutting speeds. The processing parameters such as rotation speed, rate of feed, and SiC weight percentage are used to calculate trust force, surface waviness, burr height, and wear in the tool wear and also conducted a desirability function analysis to optimize the variables. During the process, the minimum thrust force of 84 N, surface roughness of 1.671 m, and the burr height of 0.16 mm [9].

WEDM experiments use Ti–6Al–4V material to evaluate MRR and energy consumption using pulse on time, voltage, and wire feed rate. Applying a linear equation of observed data to reflect the relationship between descriptive and dependent variables yields regression models. The results show that there is an improvement in composite desirability by 7.88% at the optimum parameter [10]. Response surface methodology is used to reduce MRR and surface smoothness. The author found that the multiple responses desirability strategy improved the result [11]. Taguchi’s L27 orthogonal array technique investigates Al-15%–SiCp metal matrix composite. The DFA analysis was utilized to improve the surface quality and power usage [12]. The electrical discharge machining method is used. The Al 6061 alloy is machined by using the machining process. Process parameters include discharge current, powder percentage, pulses on time, pulse off period, and magnetic field strength. The experimental design was done by using RSM, and the process variables were optimized by using DFA. The results of the trials revealed that the discharge current is the most relevant parameter. The optimum process parameters for minimal overcut (OC) is 0.0801 mm was observed with a desirability index of 0.998 [13].

### 1.1. Response Surface Methodology

When two or more quantitative factors are present, RSM is used to improve the response. The dependent variables are replies in the response surface approach, whereas the independent variables or components are referenced as predictor variables. Turning AISI 6061 T6 aluminium was subjected to an experimental investigation to maximize energy usage, waviness, and MRR. The authors designed an experiment using the CCD of RSM. The optimization results reduce the energy consumption by 14.41%, and the surface roughness by 360.47% [14]. The experiment was carried out on LM 25 aluminium alloy utilizing the response surface approach to reduce wear in tool and surface waviness. The surface roughness is considerably affected by BUE formation at low speeds [15]. Natural aluminium is evaluated for the turning process by taking speed, cutting depth, and SiCP weight percent into account. The author employed the ANOVA and RSM approach to optimize tangential, axial, and radial cutting force. Cutting speed is the most noteworthy factor inducing the response variables [16]. To investigate the effect of cutting parameter settings on alumina ceramic material, the turning depth of penetration and surface quality are measured by using an abrasive water jet machine. Response surface methodology experiments were used to develop quadratic regression models. The optimal process conditions would lead to the maximum DOP at 390 μm and a Ra at 5.3 μm. [17]. Knowing the characteristics of surface and wall thicknesses of AA5052 Al alloys was optimized by using RSM and ANOVA [18]. A minimum surface roughness is 2.45 μm and maximum thickness of 0.753 mm, obtained at a spindle speed of 1931 rpm, feed of 654 mm/rev, and step size of 0.65 mm. A study was carried out to ascertain the effect of nose radius on surface waviness while turning aluminium (6061). RSM and ANOVA methodology is used for optimization. If the increase in cutting speed is within the specified range, it depreciates the surface finish [19]. A typical lathe machine executes a turning operation on aluminium 6061 material. The cutting depth, rate of feed, and speed are all considered while measuring cutting forces, surface waviness, and temperature by using RSM and ANOVA. The minimum cutting force, surface roughness and temperature are obtained at 7 kgf, 2.4 mm, and 58 °C for a depth of cut of 0.5 mm, cutting speed of 900 rpm, and federate of 0.2 mm [20]. The experiments are done with the help of the DOE. The trials are carried out on a CNC turning machine made of 7075 Al alloy, with speed, feed, cutting depth, and tool nose radius considered to reduce power consumption and increase the tool’s life. The DFA study was carried out by utilizing RSM, and the results were highlighted by a 13.55% reduced power consumption and a 22.12% improvement in tool life [21].

### 1.2. Desirability Function Analysis—DFA

The desirability function technique is a popular MRO strategy. The desirability scale ranges from 0 to 1, showing how near the response is to its ideal value. The numerical optimization identifies a position where the desirability function was maximized. To enhance machined settings for turning GFRP pipes with a K20 grade cemented carbide cutting tool, research known as the DFA methodology was used. To optimize surface waviness, wear on the tool, and machining power [22]. The researcher in [23] optimized the tool’s surface quality, MRR, and wear while turning the GFRP composite using DFA. The Taguchi approach and DFA methodology were used to optimize both the cutting parameters during the turning of Inconel 718 super alloy material and the ANOVA that was used to assess the impact of essential factors such as surface waviness and MRR [24]. Experiments are carried out by using the Taguchi approach in conjunction with fuzzy logic based on the desired function of bovine femur material to optimize temperature, force, and surface roughness [25].

The predictions of machining operations are crucial and depend on machining variables such as cutting speed, rate of feed, cutting depth, and tool nose radius. Limited research is available to optimize temperature by using ANOVA, RSM, and DFA approaches while machining aluminium 6061 material. This study acknowledges the temperature prediction and the factors affecting the turning operation. It aims to develop temperature prediction models while conducting turning operations as a function of several machining variables because these are critical issues to the industrial experts to reduce unnecessary heat generated in workpieces and tools.

## 2. Experimentation Design

The RSM is the most effective tool for analyzing factorial trial outcomes. RSM is a convenient tool in the engineering sector for problem-solving, analysis, and modeling, and it also gives appropriate information with lesser experiments [26,27]. The response temperature (T_ob_) is represented as a process variable function given as

Temperature Rise = ϕ(V_c_, F_z_, D_c_, R_n_) + e_ui_

where, ϕ is the response surface, e_u_ is the residual, u is the number of observations in the factorial experiment, and iu reflects the level of the ith factor in the u_th_ observation. When the mathematical form of ϕ is unknown, polynomials expressed in the form of the processing parameters variable can be utilized to estimate this function within the experimental region correctly.

The CCD methodology is the most commonly used in response to surface-planned tests. CCD designs consist of center and star points. The input parameter and their ranges are described by using the Hindustan Machine Tools data book [28,29]. Table 1 shows all four variables’ higher (+2) and lower (−2) values. Interpolation was used to compute the intermediate levels of 0 for all variables. The output response is the workpiece temperature. The design matrix utilized to execute the experiments, a four-factor central composite rotatable design with 30 coded conditions, is shown in Table 2.

### 2.1. Experimental Set-Up

**Figure 1:**
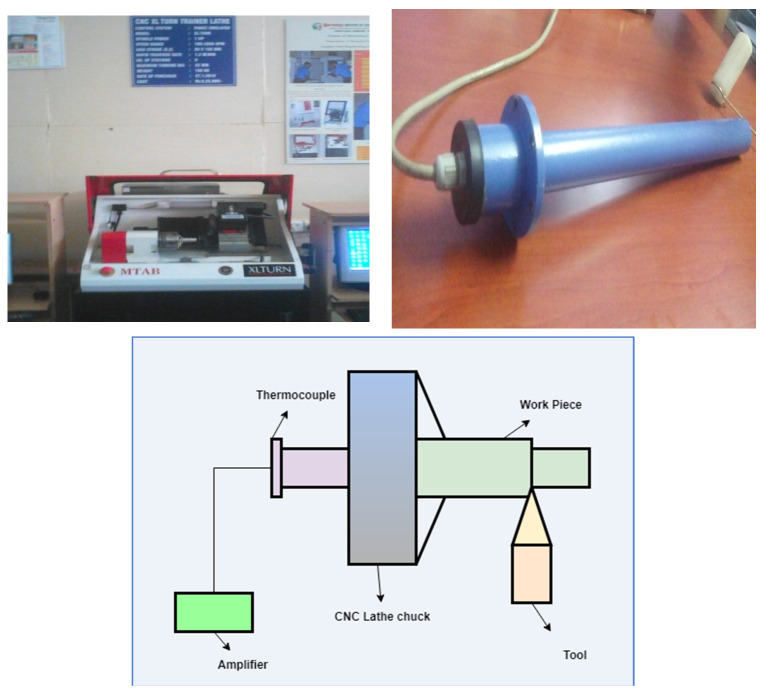
Experimental setup.

The trials were carried out on an XLTURN-CNC lathe, and Al 6061 was used as working material. In this experiment, test samples with a diameter of 40 mm and length of 100 mm were employed, and hardness was tested and was found to be 43 HRC. The tests were performed in dry circumstances with an Al_2_O_3_ coated carbide cutting tool by using a Sandvik Coromant T-Max P Turning Tool Holder (Sandviken, Sweden). Cutting speed, rate of feed, cutting depth, and tool nose radius were the machining parameters. A 1-mm hole was drilled in the workpiece at 10 mm below the machining surface and the temperature is measured by using a K-type thermocouple (illustrated in Figure 1). Table 1 shows the input parameters and their levels according to the Hindustan Machine Tools data book, and Table 2 summarizes the experiment output temperature data.

### 2.2. Temperature Prediction Using a Response Surface Model

The generalized quadratic polynomial expression for the correlation between the response surfaces y and the process parameter x is given as

Y = β_0_ + β_1_x_1_ + β_2_x_2_ + β_3_x_3_ + β_11_x_12_ + β_22_x_22_ + β_12_x_1_ (1)

where β_0_ is the constant, β_1_, β_2_, β_3_ is the linear term coefficient, β_11_, β_22_ is thequadratic term coefficient, and β_12_ is the interaction term coefficient.

The observed reading was accurately analyzed by using the Design Expert V11 software (Statease, Minneapolis, MN, USA). A second-order quadratic model was developed for temperature prediction, and the model’s relevance was validated by using an analysis of variance. The ANOVA analysis used for temperature prediction is shown in Table 3. The model’s F-value of 786.09 in Table 3 indicates that it is significant. An F-value that is this high might arise owing to noise just 0.01% of the time.

Model terms with *p*-values less than 0.0500 are significant. Significant model terms include V_c_, F_z_, D_c_, R_n_, V_c_ D_c_, V_c_R_n_, F_z_D_c_, F_z_R_n_, D_c_R_n_, V_c_^2^, F_z_^2^, D_c_^2^, and R_n_^2^. The presence of values larger than 0.1000 suggests that the model terms are insignificant. Model reduction may enhance the model if there are numerous inconsequential model terms (except those necessary to enable hierarchy).

The F-value for lack of fit of 0.2759 indicates that the lack of fit is slight compared to the pure error. A lack of fit F-value that is this big is almost certainly due to noise, and a non-significant lack of fit is desirable. The regression equation generated by Design Expert V11 software is shown below.

Temperature = +123.32917 − 1.86083 × V_c_ − 19.67593 × F_z_ + 29.35417 × D_c_ − 43.10417 × R_n_ − 0.023148 × V_c_ × F_z_ − 0.260417 × V_c_ × Dc + 0.431250 × V_c_ × R_n_ − 23.26389 × F_z_ × D_c_ + 23.95833 × F_z_ × R_n_ − 28.59375 × D_c_ × R_n_ + 0.009097 × V_c_^2^ + 44.36728 × F_z_^2^ + 14.60937 × D_c_^2^ + 7.42187 × R_n_^2^ (2)

In this work, the input parameters were used to develop mathematical models by using the DOE and RSM approaches. As shown in Table 3, the estimated F-ratio is greater than the traditional (tabulated) F-ratio for temperature rise, suggesting that the model meets the required 95% confidence level. The gap between experimental and anticipated values is within acceptable bounds.

## 3. Results and Discussions

### 3.1. Interaction Effect

**Figure 2:**
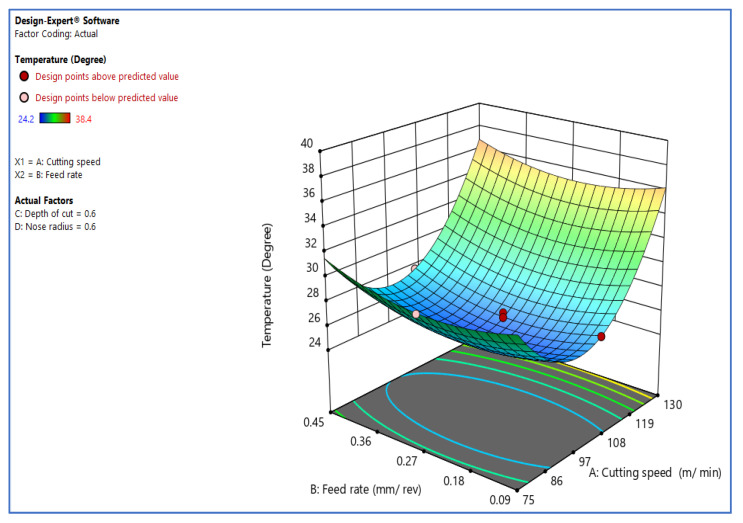
Inter. effect of V_c_ vs. F_z_ over temp.

This section discusses the interaction impact of the process factors on temperature rise. Figure 2 depicts the cutting speed’s interaction effect against the rate of feed on temperature. The above interaction diagram indicates that the cutting speed and rate of feed significantly impact the turning process’s temperature increase. The graph demonstrates that increasing the cutting speed [6] induces a temperature increase, as does decreasing the cutting speed, which is minor for cutting speeds ranging from 91 to 102 m/min. As the cutting speed increases, the rate at which energy is lost caused by plastic distortion and friction increases, and tool life will be reduced if cutting speed increases. The tool’s cutting edge will crumble if the cutting speed is too slow or too quick. Increases in rate of feed, cutting temperature, and flank wear all directly affect the material’s surface and tend to make it wavier.

**Figure 3:**
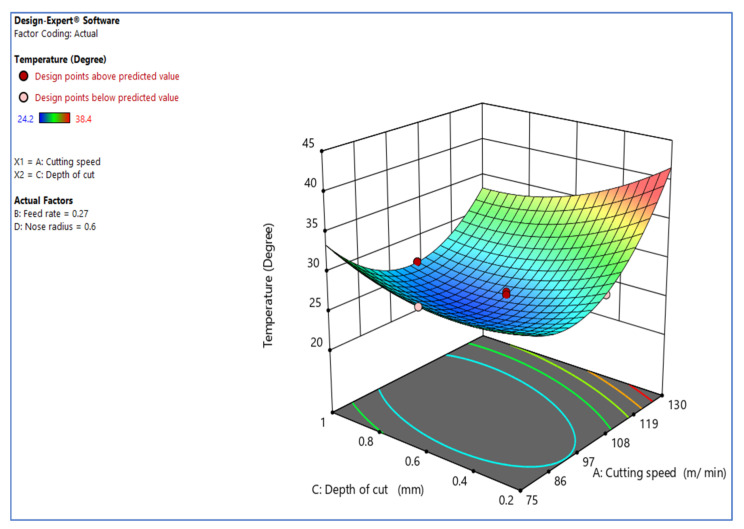
Inter. effect of V_c_ vs. D_c_ over temp.

Figure 3 shows how the cutting speed against the cutting depth [6] significantly affects the turning process’s temperature. The graph indicates that raising the cutting depth of the incision causes the temperature to rise. The quantity of removed workpiece material rises with cutting depth and temperature. Less workpiece material clings to the tool’s side at lesser depths of cut than at more considerable cutting depth. The temperature rises due to the adhesion of the work material to the tool flank. The ANOVA table confirms the results.

**Figure 4:**
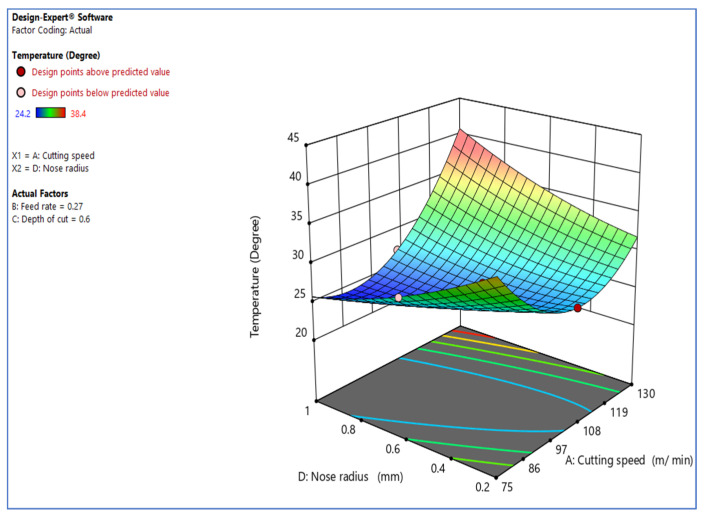
Inter. effect of V_c_ vs. R_n_ over temp.

Figure 4 depicts the interaction and effect of cutting speed vs. tool nose radius [19] on temperature rise. The interaction diagram reveals that the tool nose radius significantly impacts the temperature rise throughout the turning process. According to the graph, lowering the tool nose radius minimizes the temperature rise. The ANOVA table further validates the findings. The tool nose radius primarily influences the cutting temperature; the more significant the tool nose radius, the greater the distortion and cutting conditions, and the more heat produced during chip creation. Increasing the tool nose radius, on the other hand, elongates the working portion of the leading edge and raises the mass of the tooltip.

**Figure 5:**
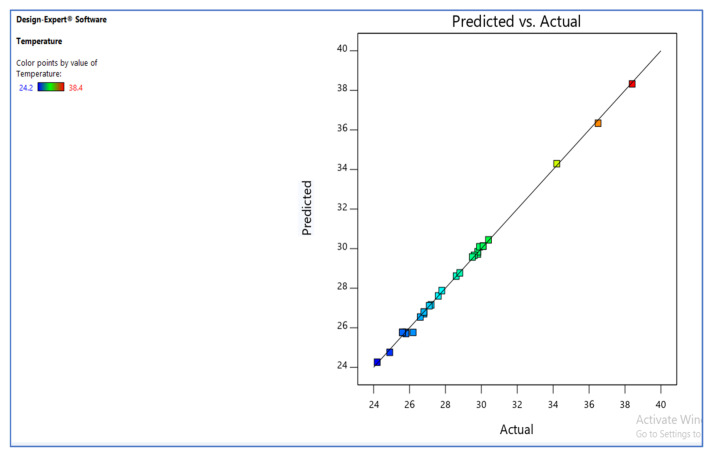
Predicted vs. actual.

The graph of observed values vs. expected values is shown in Figure 5, which aids in detecting observations that the model fails to predict. The 45° line should split the data points evenly [29].

### 3.2. Implementation of DFA

In this study, the quality aspects of the turning process were assessed by employing cutting parameters to optimize temperature throughout the cutting operation. The smaller-the-better-type desirability function was used for temperature rise to normalize their values and assess individual desirability indices in the range of [0, 1] [30]. According to Harrington [31], Equation (3) establishes the smaller-the-better quality characteristic used when the response is lowered. y_tgt_ is the response’s lowest value in this situation. Then we have

y_i_, i.e., y_tgt_ = y_min_.

(3) $$\mathrm{di}=\{\begin{array}{c} 1\\ 0 \end{array}\;{\left(\frac{y_{i\;-}y_{\max}}{y_{\mathrm{tgt}}-{\;y}_{\max}}\right)}^{r}\;$$

where, $y_{i}<y_{\mathrm{tgt}}$; $y_{\mathrm{tgt}}<y_{i\;}<y_{\max}\;,\;r\geq 0\;$ and $y_{i}<y_{\max}$.

As a result, Equation (3) is adjusted as given in Equation (4),

(4) $$\mathrm{Di}=\{\begin{array}{c} 1\\ 0 \end{array}\;{\left(\frac{y_{I\;-}y_{\max}}{y_{\min}-{\;y}_{\max}}\right)}^{r}\;$$

where, $y_{i}<y_{\min}$; $y_{\min}<y_{i\;}<y_{\max}\;,\;r\geq 0\;$ and $y_{i}<y_{\max}$.

The most desired circumstance in this research is when y_i_ is smaller than y_min_, and the individual desirability index becomes 1. When y_i_ deviates from y_min_, the value of di falls and equals 0 when y_i_ exceeds y_max_. The di values produced are in the [0, 1] in all other cases. Depending on their respective relevance, the weights allocated to each reply are represented by the exponent’s r. Equation (5) can be used to compute the geometric mean of the individual desire as the composite desirability for each experimental circumstance. Then we have

(5) Composite Desirability (CD)=(d1w1 × d2w2 × d3 w3 x … … … )1k

where, k is the number of responses, d_1_, d_2_, d_3_ are the individual desirability indices, w_1_, w_2_, w_3_ are the weight-assigned responses, and w = $\sum_{i}^{k}w_{i\;}=1$.

If any of the replies is entirely unacceptable, the value of CD becomes 0, i.e., d_i_ = 0. Individual desirability of temperature rise is calculated by using the desirability functions given in Equation (6). Temperature increase is given equal weightage (w_1_ = w_2_ = 1). Then we have

(6) $$d_{\mathrm{temp}}=\{\begin{array}{c} 1\\ 0 \end{array}\;{\left(\frac{y_{i\;}-38.8}{24.2-38.8}\right)}^{1}$$

where, $y_{i}<24.2;$ $24.2<y_{i\;}<38.8,\;r\geq 0\;$ and ${\;y}_{i}<38.8$.

Equation (7) was used to calculate the temperature rise based on the answers under examination:

(7) CD=(dtemp1 )12 i=1 to 30

The best parameter circumstances have the highest overall desirability (TD) factor settings. The concurrent objective function is the geometric mean of all changing responses. Design Expert software analyzes the combination based on the composite desirability optimization approach. Models have been created to optimize temperature increases. A measure of how the solution has achieved the needed objectives for all the answers must be assessed in the multi-response optimization (MRO). To limit temperature rise, the best approach is to check the process input parameters. Table 4 and Table 5 show the optimum parameter combination values and the projected replies’ projected values.

**Figure 6:**
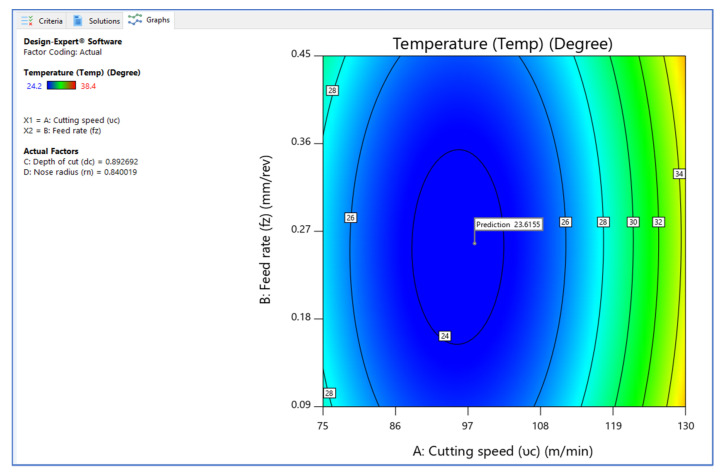
Contour plot on desirability (V_c_ vs. F_z_).

**Figure 7:**
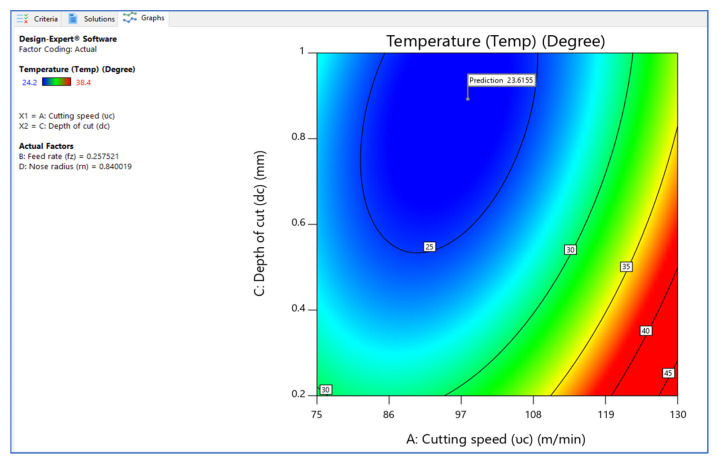
Contour plot on desirability (V_c_ vs. D_c_).

**Figure 8:**
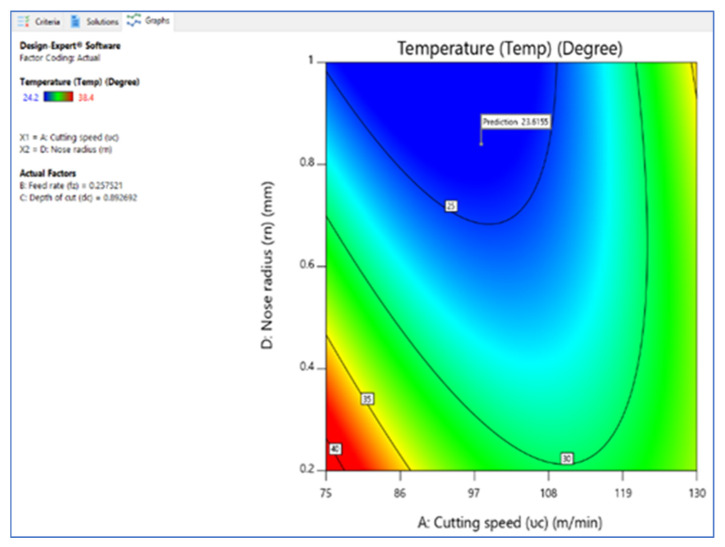
Contour plot on desirability (V_c_ vs. R_n_).

Figure 6, Figure 7 and Figure 8 illustrate the desirability contour plot that show the relation between the cutting speed and the rate of feed over temperature variable. This plot in Figure 6 shows values of Z variables to X and Y variables. The link between the three variables was investigated by using these contour plot diagrams. The *x*- and *y*-axes show two independent variables, whereas the *z*-axis shows one dependent variable. Contour plots aid in the identification of combinations that provide favorable result values. Similarly, Figure 7 and Figure 8 depict the relationship of the cutting speed with the cutting depth and nose radius over temperature, respectively.

**Figure 9:**
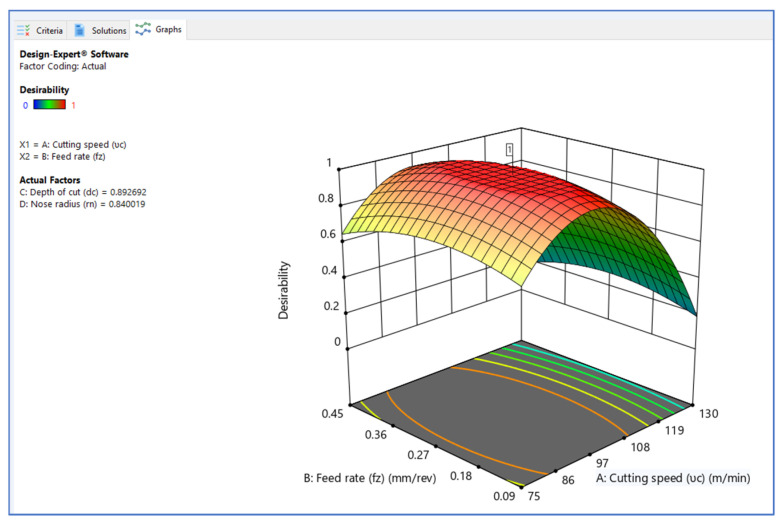
Surface plot on desirability (V_c_ vs. F_z_).

**Figure 10:**
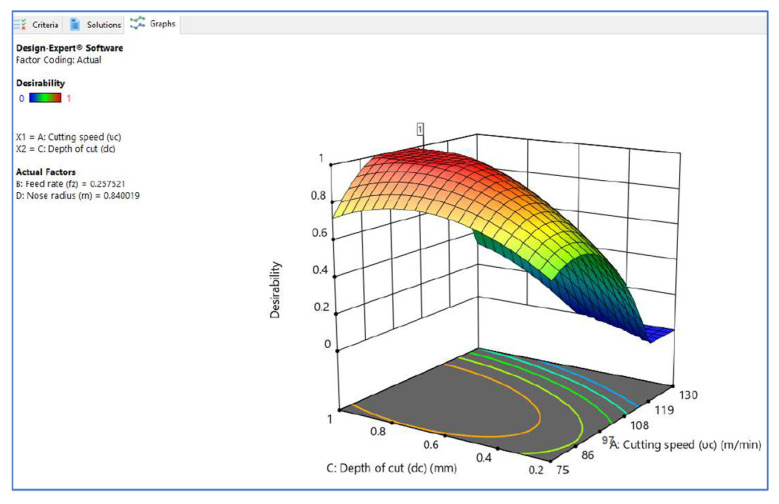
Surface plot on desirability (V_c_ vs. D_c_).

**Figure 11:**
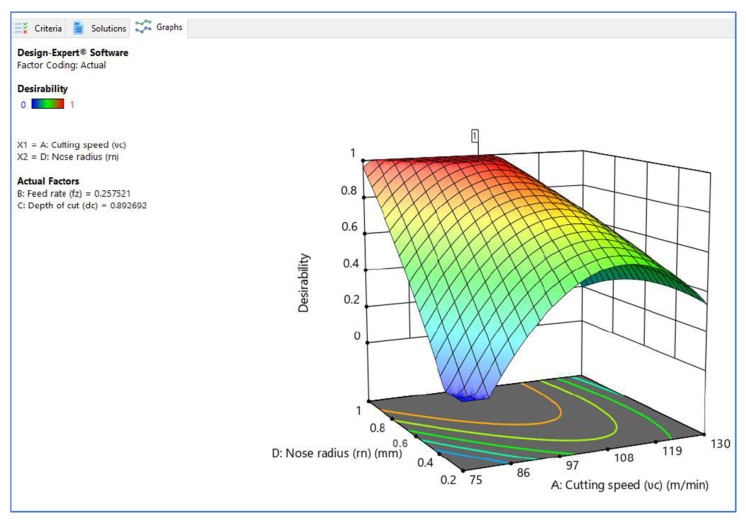
Surface plot on desirability (V_c_ vs. R_n_).

**Figure 12:**
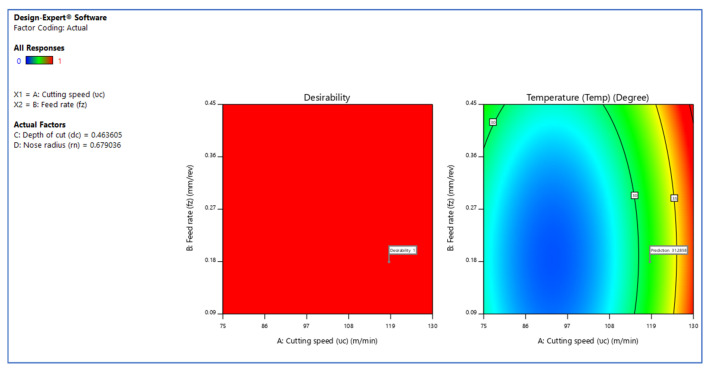
Contour plot of the variable on temperature.

The surface plot on desirability is shown in Figure 9, Figure 10, Figure 11 and Figure 12. The plot in Figure 9 shows the surface plot between cutting speed and feed rate over desirability value, which is close to 1. The surface plots are three-dimensional data visualizations. A surface plot is a two-dimensional representation of a three-dimensional connection, with variables on the *x*- and *y*-axes and a smooth texture representing the dependent variables on the *z*-axis. As given in the plots of Figure 9, Figure 10 and Figure 11, the desirability value of cutting speed with feed rate, depth of cut and nose radius are close to 1 as per DFA methodology.

**Figure 13:**
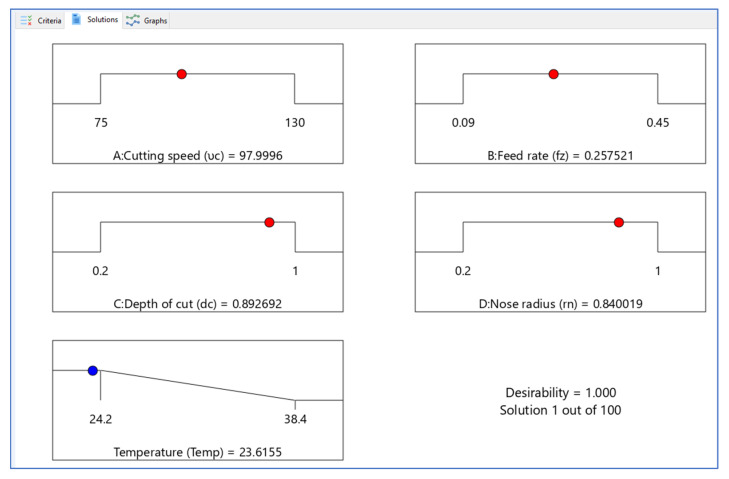
Ramp function graph of desirability.

**Figure 14:**
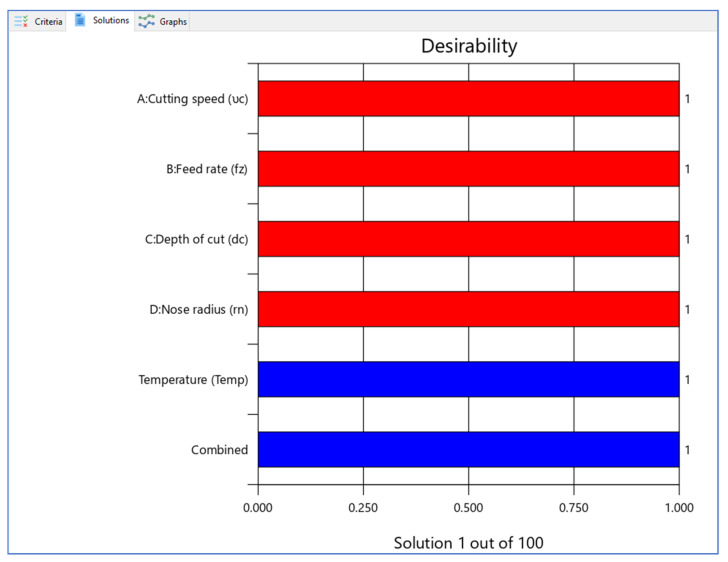
Bar graph of desirability.

Design Expert V11 software was used for optimization, and 100 solutions were obtained during optimization. The optimal combination for getting the desired reactions was a set of situations with the highest desirability value. The best combinations with higher desirability functions are shown in Table 5. Figure 13 and Figure 14 depict the ramp functions and bar graphs illustrating the desirability of output responses, respectively. The dot at the top of each ramp reflect the factor setting (or) response forecast for that response characteristic. The size of the dot reflects how much it is wanted. For instance, the red colour dots show the optimized machining parameters, i.e., 97.9996 m/min cutting speed, 0.257521 mm/rev rate of feed, 0.892692 mm cutting depth and 0.840019 mm tool nose radius are the optimized values. The blue colour dots show the optimized predicted temperature, which is 23.615 °C. When the weight for each parameter was set to one, a linear ramp function is generated between the lower and required values (or the higher and required values). The bar graph depicts the overall desirability of the responses. The optimal zone has an overall desirability value of 23.615 °C, suggesting it is close to the target response.

## 4. Validation of the Model

The desirability function analysis predicted the optimal conditions, which was validated by physical measurements. The model’s validity is demonstrated because the error percentage is less than ±2%. Surface roughness and temperature testing findings indicate good agreement with the optimal cutting settings (as predicted by DSA) as shown in Table 6.

## 5. Conclusions

Due to the critical machining properties, the experimentation to predict temperature rise was done using Al 6061 workpiece material and Al_2_O_3_ carbide coated cutting tool. The input parameters considered were cutting speed, rate of feed, cutting depth, and tool nose radius. The experiments were designed by using design of experiment (DoE)-based response surface methodology and the desirability function approach. The mathematical model was developed to verify for its adequacy by using analysis of variance (ANOVA). The ANOVA table showed that the model value is less than 0.0001, implying that the model is significant, and the F-value for lack of fit is 0.2759, indicating that it is not significant due to noise.

The interaction effect diagram of RSM methodology revealed that cutting speed is the most significant factor, followed by rate of feed, cutting depth, and tool nose radius. This shows that the cutting speed is the stimulating factor of temperature rise throughout the turning process and the temperature rise is minimal between 91 m/min and 98 m/min of cutting speed, 0.6 mm and 0.8 mm of nose radius. Therefore, increasing cutting speed dramatically increases the temperatures of the workpiece. Furthermore, multi-response optimization by desirability analysis shows that the minimum value of temperature rise parameters are 98.0 m/min cutting speed, 0.258 mm rate of feed, 0.893 mm cutting depth, and 0.84 mm tool nose radius. The optimal zone has an overall desirability rating of 23.615 °C, indicating that it is close to the desired response. The models are validated, and the error percentage is less than ±2%. Temperature rise outcomes indicate that the experiments have good agreement with the optimal cutting settings.

## Figures and Tables

**Table 1:** The levels of process parameters.

Sl. No	Parameter Considered	Factorial Levels				
		−2	−1	0	1	2
1	Cutting speed, V_c_ (m/min)	75	90	105	120	130
2	Rate of feed, F_z_ (mm/rev)	0.09	0.18	0.27	0.36	0.45
3	Cutting depth, D_c_ (mm)	0.2	0.4	0.6	0.8	1.0
4	Tool Nose radius, R_n_ (mm)	0.2	0.4	0.6	0.8	1.0

**Table 2:** Responses to experimental values.

Sl. No	V_c_ (m/min)	F_z_ (mm/rev)	D_c_ (mm)	R_n_ (mm)	Temperature Rise °C T_ob_ (Observed)	Temperature Rise °C (T_RSM_) (Pred. by RSM)
1	120	0.36	0.8	0.4	28.8	28.77
2	90	0.18	0.8	0.4	29.8	29.84
3	105	0.27	1.0	0.6	26.6	26.55
4	105	0.27	0.6	0.6	25.8	25.77
5	90	0.36	0.8	0.8	24.9	24.76
6	105	0.27	0.6	0.2	26.8	26.80
7	120	0.36	0.8	0.8	29.7	30.10
8	105	0.27	0.6	0.6	28.2	25.77
9	75	0.27	0.6	0.6	29.3	29.58
10	90	0.18	0.4	0.4	27.1	26.71
11	90	0.36	0.8	0.4	28.6	28.61
12	105	0.27	0.6	0.6	25.6	25.77
13	105	0.27	0.2	0.6	29.6	29.66
14	120	0.18	0.8	0.8	29.8	29.73
15	120	0.18	0.4	0.4	30.1	30.12
16	105	0.27	0.6	0.6	25.6	25.77
17	90	0.36	0.4	0.8	27.8	27.88
18	90	0.36	0.4	0.4	27.2	27.16
19	105	0.27	0.6	1.0	27.1	27.11
20	120	0.18	0.4	0.8	34.2	34.30
21	90	0.18	0.4	0.8	25.8	25.71
22	105	0.45	0.6	0.6	27.6	27.61
23	105	0.09	0.6	0.6	26.8	26.80
24	120	0.36	0.4	0.4	30.4	30.45
25	105	0.27	0.6	0.6	25.8	25.77
26	120	0.18	0.8	0.4	30.1	30.13
27	135	0.27	0.6	0.6	38.8	38.33
28	90	0.18	0.8	0.8	24.2	24.26
29	120	0.36	0.4	0.8	36.5	36.34
30	105	0.27	0.6	0.6	25.6	25.77

**Table 3:** ANOVA table for temperature rise prediction.

Source	Sum of Squares Value	df	Mean Square Value	F-Value	*p*-Value	
Model	311.21	14	22.23	786.09	<0.0001	significant
V_c_	49.56	1	49.56	1752.53	<0.0001	
F_z_	1.01	1	1.01	35.72	<0.0001	
D_c_	9.70	1	9.70	343.14	<0.0001	
R_n_	0.4290	1	0.4290	15.17	0.0014	
V_c_ F_z_	0.0156	1	0.0156	0.5526	0.4688	
V_c_ D_c_	9.77	1	9.77	345.35	<0.0001	
V_c_ R_n_	26.78	1	26.78	947.06	<0.0001	
F_z_ D_c_	2.81	1	2.81	99.22	<0.0001	
F_z_ R_n_	2.98	1	2.98	105.23	<0.0001	
D_c_ R_n_	20.93	1	20.93	740.18	<0.0001	
V_c_^2^	114.92	1	114.92	4063.88	<0.0001	
F_z_^2^	3.54	1	3.54	125.27	<0.0001	
D_c_ ^2^	9.37	1	9.37	331.24	<0.0001	
R_n_^2^	2.42	1	2.42	85.49	<0.0001	
Residual	0.4242	15	0.0283			
Lack of Fit	0.1508	10	0.0151	0.2759	0.9606	Insignificant
Pure Error	0.2733	5	0.0547			
Cor Total	311.63	29				

**Table 4:** Selection of factors and responses for desirability.

Sl. No	Input Parameter	Goal	Lower Limit	Upper Limit
1	V_c_	In range	75	130
2	F_z_	In range	0.09	0.45
3	D_c_	In range	0.2	1.0
4	R_n_	In range	0.2	1.0
5	Temperature rise Observed (T_ob_)	Minimize	24.2	38.8

**Table 5:** Optimum values during turning of aluminium.

Sl. No	Input Parameter	Goal	Optimum Value
1	Vc	In range	98.0 (m/min)
2	Fz	In range	0.26 (mm/rev)
3	Dc	In range	0.893 (mm)
4	Rn	In range	0.84 (mm)
5	Temperature rise Observed (Tob)	Minimize	23.615 °C

**Table 6:** Optimized process parameter—validation model.

Sl. No	V_c_	F_z_	D_c_	R_n_	Confirmatory Test (Temp., °C)		% Error
					Optimum Value by DFA	Exp. Value	
1	98.0	0.258	0.893	0.84	23.615	23.610	0.021
2	90.3	0.136	0.654	0.86	23.943	23.896	0.196
3	94.7	0.199	0.730	0.78	24.098	24.061	0.154

## Data Availability

The data supporting this study’s findings are available upon request from the authors.
